# Characterization of *Bacillus anthracis *arginase: effects of pH, temperature, and cell viability on metal preference

**DOI:** 10.1186/1471-2091-9-15

**Published:** 2008-06-03

**Authors:** Ryan J Viator, Richard F Rest, Ellen Hildebrandt, David J McGee

**Affiliations:** 1 Department of Biology, University of South Alabama, Mobile, AL 36688, USA; 2 Department of Microbiology and Immunology, Drexel University College of Medicine, Philadelphia, PA 19129, USA; 3 Department of Microbiology & Immunology, Louisiana State University Health Sciences Center, Shreveport, LA 71130, USA

## Abstract

**Background:**

Arginase (RocF) hydrolyzes L-arginine to L-ornithine and urea. While previously characterized arginases have an alkaline pH optimum and require activation with manganese, arginase from *Helicobacter pylori *is optimally active with cobalt at pH 6. The arginase from *Bacillus anthracis *is not well characterized; therefore, this arginase was investigated by a variety of strategies and the enzyme was purified.

**Results:**

The *rocF *gene from *B. anthracis *was cloned and expressed in *E. coli *and compared with *E. coli *expressing *H. pylori rocF*. In the native organisms *B. anthracis *arginase was up to 1,000 times more active than *H. pylori *arginase and displayed remarkable activity in the absence of exogenous metals, although manganese, cobalt, and nickel all improved activity. Optimal *B. anthracis *arginase activity occurred with nickel at an alkaline pH. Either *B. anthracis *arginase expressed in *E. coli *or purified *B. anthracis *RocF showed similar findings. The *B. anthracis *arginase expressed in *E. coli *shifted its metal preference from Ni > Co > Mn when assayed at pH 6 to Ni > Mn > Co at pH 9. Using a viable cell arginase assay, *B. anthracis *arginase increased dramatically when the cells were grown with manganese, even at final concentrations of <1 μM, whereas *B. anthracis *grown with cobalt or nickel (≥500 μM) showed no such increase, suggesting existence of a high affinity and specificity manganese transporter.

**Conclusion:**

Unlike other eubacterial arginases, *B. anthracis *arginase displays unusual metal promiscuity. The unique properties of *B. anthracis *arginase may allow utilization of a specific metal, depending on the *in vivo *niches occupied by this organism.

## Background

*Bacillus anthracis *is a highly invasive, exceptionally virulent pathogen of mammals, with humans as accidental hosts [1]. *B. anthracis *possesses two virulence plasmids, pXO1 and pXO2, that encode for a tripartite secreted toxin and a poly-D-glutamic acid capsule, respectively [2-4]. The toxins can cause death or produce serious edema in infected individuals [5,6]. Despite many years of research, chromosomal loci have received little attention; however, a chromosomally-encoded cytotoxin, anthrolysin O, has been recently reported [7].

Arginase hydrolyzes L-arginine to L-ornithine and urea. This enzyme is found in both eubacteria and eucaryotes. However, relatively few eubacterial arginases have been characterized; most characterized arginases are from animals and yeasts [8-10]. We recently characterized an arginase (RocF) from the gastric pathogen, *Helicobacter pylori *[11], discovering a number of interesting and unique properties among the arginase superfamily. When we began this current study, the closest homolog of the *H. pylori *arginase (NCBI Accession Number: NP_208190) was that from *B. anthracis *(NCBI Accession number: YP_016761 [12]. Yet, the two RocF proteins share only 20% amino acid identity over the entire protein (27% when comparing the first 283 amino acids of *H. pylori *with the first 271 amino acids of *B. anthracis*). Arginase activity from *H. pylori *has recently been measured using a highly sensitive and quantitative assay that can determine enzyme activity from extracts, viable cells or the purified enzyme [11]. The *rocF *gene from *H. pylori *has been cloned into *E. coli *DH5α and confers arginase activity to *E. coli *(which does not possess a native arginase), suggesting that the *rocF *gene alone is sufficient to confer arginase activity [11].

L-arginine is used by macrophages to produce nitric oxide and other downstream reactive nitrogen species [13]. The production of nitric oxide by activated host macrophages is an effective antimicrobial agent and serves as an initial innate defense mechanism against pathogens [13]. Interestingly, the *H. pylori *arginase inhibits host nitric oxide production, allowing for survival of the organism when co-cultured with activated macrophages [14]. A similar situation likely occurs with the *B. anthracis *enzyme [15]. Additionally, *H. pylori *arginase decreases T lymphocyte proliferation and CD3ζ expression [16], arguing for the importance of this enzyme in multiple facets of host-pathogen interactions.

In addition to roles in pathogenesis, it is speculated that arginases have evolved to regulate the levels of arginine and ornithine within the cell [17]. Regulation of these amino acids is pivotal in protein synthesis, polyamine and nitric oxide production, and other cellular processes [17]. For example in *H. pylori*, expression of a second copy of the arginase gene, *rocF*, does not double the arginase activity, suggesting the bacterium has mechanisms to limit hydrolysis of the essential amino acid, arginine [18].

In organisms having a complete urea cycle, arginase is the final enzyme of the cycle; however, arginase is not found in most eubacteria [19]. In *H. pylori*, the urea is further hydrolyzed into carbon dioxide and ammonia via the enzyme urease [20]. Most eubacteria that contain an arginase also have the entire urea cycle. Examples include *H. pylori *and *B. subtilis *[20,21]. However, several arginase-containing bacteria apparently lack a complete urea cycle. Such organisms include *B. licheniformis *[22] and *B. anthracis*. This suggests that arginase in these latter bacteria has evolved a unique physiologic role in the cell. Several biochemical properties of the *B. anthracis *arginase had been preliminarily described [23]. Purified *B. anthracis *arginase had to be preactivated with 1 μM Mn^2+ ^(MnSO_4_) and heat (37°C for 3 hours) (designated as heat-activation) for activity to be measured using a urea detection method [23]. Indeed, nearly all arginases require some type of heat activation step in the presence of manganese for catalytic activity [24]. For *B. anthracis *arginase, manganese was previously considered the most efficient cation activator and stabilizer, allowing the Mn^2+^-preactivated enzyme to maintain stability and activity even after exposure to 50–60°C for one hour [23]. Additionally, the enzyme showed optimal catalytic activity between pH 9.8 and 10.0 [23], a finding consistent will all arginases [25], except the *H. pylori *enzyme (see results) [11].

Activity of the purified *B. anthracis *arginase decreased significantly after dialysis and lyophilization; if the manganese-preactivated enzyme was treated with other divalent cations, a large decrease in arginase catalytic activity occurred [23]. Collectively, these earlier data suggested that the metal cofactor involved in *B. anthracis *arginase activity was manganese [23], with nickel and cobalt having no role or an inhibitory role. The earlier data also indicated that heat-activation was absolutely required for catalytic activity. Identification of the gene responsible for *B. anthracis *arginase was not previously reported.

While the toxin components produced by *B. anthracis *have been the primary focus of research, there is limited research on the organism's other genes and gene products. In this study, we sought to characterize in depth the chromosomally-encoded *B. anthracis *arginase using a variety of conditions not previously examined, using a sensitive enzyme assay. We also established an *E. coli *model for the *B. anthracis *arginase and determined whether arginase could be detected and characterized from viable organisms. We provide evidence that the enzyme has novel features not previously recognized. For example, the *B. anthracis *arginase displayed optimal activity with nickel in extracts and manganese in viable cells, but surprisingly did not absolutely require either heat activation or the addition of exogenous metal to detect arginase activity. The *B. anthracis *arginase gene could complement an *H. pylori *arginase mutant, conferring *B. anthracis *arginase-like properties to *H. pylori*.

## Results

### Characterization of arginase-containing extracts of *B. anthracis *7702

**Figure 1:**
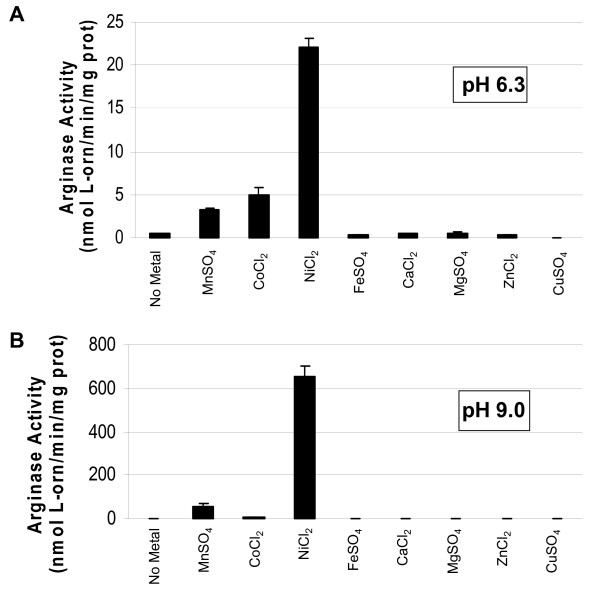
***B. anthracis *7702 extracts show optimal catalytic activity with nickel**. Cells were grown on CBA plates at 37°C for 24 h in 5% CO_2 _in air, harvested, and extracts prepared as described in Methods. The extracts were assayed with 5 mM (final concentration) of the appropriate metal after heat activation at pH 6.3 (A) or 9.0 (B) (Bis Tris Propane [BTP]-buffered arginine).

Many holes remain in our knowledge of the chromosomally-encoded arginase from *B. anthracis*. Also, no two eubacterial arginases have ever been directly compared for their properties under identical conditions. We therefore sought to investigate the *B. anthracis *arginase in much more detail and compared it to the *H. pylori *arginase. Extracts from *B. anthracis *7702 assayed with manganese (Mn^2+^) or cobalt (Co^2+^) displayed arginase activity at both pH 6.3 and 9.0; however, when assayed in the presence of nickel (Ni^2+^) at pH 9.0, the activity was up to 500-fold higher than with Mn^2+ ^or Co^2+ ^(Fig. 1A &1B). This is in striking contrast to what was reported previously for this enzyme, in which Ni^2+ ^inhibited activity [23].

**Figure 2:**
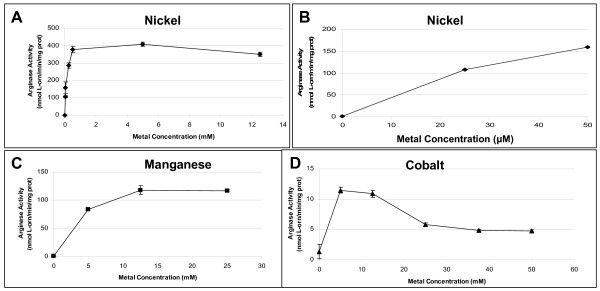
**Effect of metal concentration on arginase activity in *B. anthracis *7702 extracts**. Cells were grown on CBA plates at 37°C for 24 h in 5% CO_2 _in air, harvested, and extracts prepared as described in Methods. The extracts were heat-activated (50–55°C for 30 minutes) with increasing concentrations of NiCl_2 _(A, high concentrations; B, low concentrations), MnSO_4 _(C), or CoCl_2_(D) at pH 9.0 (in BTP-buffered arginine).

*B. anthracis *arginase activity was determined at various concentrations of nickel, manganese, or cobalt. Far lower concentrations of nickel were needed to obtain abundant arginase activity, than when cobalt or manganese were used (Fig. 2). Micromolar nickel activated arginase >2-fold more than the optimal level of manganese (400 U versus 125 U) and >100-fold higher than the optimal level of cobalt (400 U versus 12 U). Optimal concentrations for the three metals were as follows: nickel, ≥ 500 μM; manganese, ≥ 12.5 mM; and cobalt 5 mM (Fig. 2A–D). Since all three metals caused near optimal arginase activity at 5 mM, this concentration was used for most subsequent experiments.

**Figure 3:**
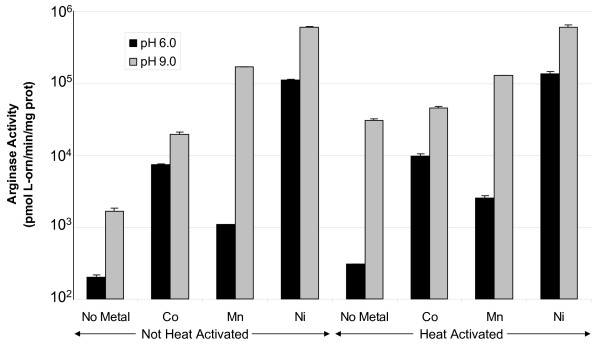
**Effect of heat activation on the arginase activity of *B. anthracis *7702 extracts**. Cells were grown on CBA plates at 37°C for 24 h in 5% CO_2 _in air, harvested, and extracts prepared as described in Methods. The extracts were assayed with or without heat activation (50–55°C or on ice for 30 minutes, respectively) in the absence (no metal) or presence of 5 mM metal (Co, Mn, or Ni) at pH 6.0 (in MES-buffered arginine) or 9.0 (Tris-buffered arginine). There was no evidence of a buffer effect on activity (data not shown).

Heat activation is required for all previously characterized arginases [24,26], presumably to allow partial unfolding of the enzyme and incorporation of the metal cofactor. In a previous study, *B. anthracis *arginase activity decreased in samples exposed to heat, if the enzyme had not been already pre-activated with manganese [23]. In contrast with this earlier finding, we observed that *B. anthracis *arginase activity surprisingly increased about 10-fold following treatment with heat (50–55°C, 30 min) at pH 9.0 in the absence of metal versus no heat (Fig. 3). However, in the presence of various metals, heat treatment did not affect arginase activity at either pH 6.0 or 9.0, when compared to samples that were not heat-treated (Fig. 3). Under all conditions, nickel conferred that highest arginase catalytic activity.

### Comparative characteristics of the *H. pylori *and *B. anthracis *arginases

**Figure 4:**
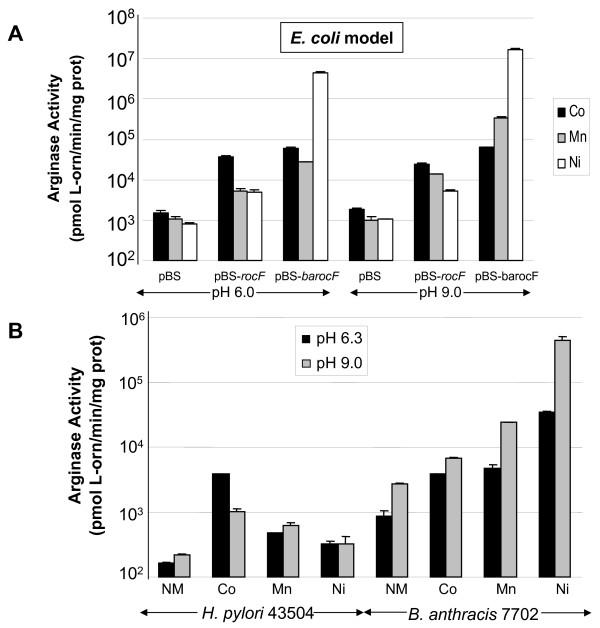
**Characteristics of arginase extracts from *E. coli *DH5α expressing the *H. pylori *or *B. anthracis *enzymes or from native organisms**. Extracts were prepared from *E. coli *carrying *H. pylori *(pBS-*rocF*) or *B. anthracis rocF *(pBS-*barocF*) and grown in L broth at 37°C with aeration (A) or from 24 hour cultures of *B. anthracis *and *H. pylori *grown on CBA at 37°C in 10% CO_2_, 5% O_2 _(B). Extracts were heat-activated for 30 minutes at 50–55°C in the presence of ddH_2_O (no metal control), 5 mM CoCl_2_, MnSO_4_, or NiCl_2 _and assayed at pH 6.0 or 9.0 in MES-buffered arginine or Tris-buffered arginine, respectively (A) or at pH 6.3 or pH 9.0 in BTP-buffered arginine (B). Arginase activity data are the mean arginase activity ± standard deviation of one representative of at least two experiments (independently prepared extracts) conducted in triplicate. Under these conditions, *E. coli *DH5α(pBS) showed a background of ~1000 pmol L-orn/min/mg protein [U]. Lack of error bars on some bars indicates that the standard deviation was minimal and did not appear on the graph. NM, no metal.

Since the *H. pylori *and *B. anthracis *arginases share low amino acid similarity (20% identity), we questioned whether the properties of these two enzymes were similar or distinct, when assayed under identical conditions. *H. pylori *and *B. anthracis *arginase activities expressed in *E. coli *DH5α were first compared (Fig. 4A). *E. coli *(pBS-*barocF*) had optimal arginase activity at both pH 6.0 and 9.0 with nickel, with lower activity in the presence of cobalt or manganese (Fig. 4A). At pH 6.0, *E. coli *(pBS-*barocF*) showed better activity with cobalt than with manganese (*p *< 0.05, Co versus Mn) (Fig. 4A). At pH 9.0, the optimal activating metal was nickel for *E. coli *(pBS-*barocF*), but higher arginase activity occurred when assayed with manganese rather than cobalt. With *E. coli *(pBS-*rocF*), optimal arginase activity was with cobalt (*p *< 0.05, Co versus Mn) (Fig. 4A) at either pH 6.0 or 9.0.

Arginase activity of *E. coli *(pBS-*barocF*) was approximately 10-fold higher at pH 9.0 than at 6.0 in the presence of manganese (Fig. 4A, *p *< 0.005). While *E. coli *(pBS-*rocF*) displayed optimal activity with cobalt when assayed at different pHs, *E. coli *(pBS-*barocF*) had optimal activity with nickel at different pHs. As previously reported [11], the *H. pylori *arginase in the *E. coli *model (pBS-*rocF*) displayed optimal catalysis with cobalt at pH 6.0.

In addition, enzyme activities from native organisms were compared after growth under identical conditions (Campylobacter blood agar, 24 h, 37°C, 5% O_2_/10% CO_2_). While the *B. anthracis *enzyme had greater activity with nickel than without metal (>50-fold difference irrespective of pH), the enzyme still surprisingly retained activity even in the absence of metal (Fig. 4B, NM). At pH 9.0 arginase activity in *B. anthracis *extracts was up to 1000-fold higher than in *H. pylori *extracts depending on the metal present and the pH of assay (Fig. 4B). Even when assayed under sub-optimal conditions (*e. g*., pH 6 with manganese), the *B. anthracis *arginase was still more activity than the *H. pylori *enzyme (Fig. 4B). The only condition in which the *H. pylori *arginase was as active as the *B. anthracis *enzyme was when the enzymes were assayed at pH 6.3 in cobalt (Fig. 4B). The results suggest that the *E. coli *models nearly completely mimic the data obtained from native organisms.

### Comparison of arginase activity between *E. coli *DH5α pBS-*barocF *and *B. anthracis *7702

To strengthen the validity of the *E. coli *arginase model, we directly compared the arginase activities between *E. coli *(pBS-*barocF*) versus *B. anthracis *under identical conditions. Under most assay conditions, the arginase expressed in *E. coli *versus *B. anthracis *showed similar trends in that highest arginase activity occurred at pH 9 with nickel (data not shown). However, arginase activity in *E. coli *(pBS-*barocF*) was 5–15-fold higher than in *B. anthracis *under most conditions, probably due to increased gene dosage from the high-copy-number plasmid. The only case in which *B. anthracis *arginase activity was not significantly lower than arginase expressed in the *E. coli *model was when the extracts were assayed without metal. The second most optimal metal shifted from manganese at pH 9.0 to cobalt at pH 6.3, especially in the *E. coli *model (data not shown). In addition, there was substantial activity without heat treatment or without addition of exogenous metal (data not shown).

### Metal dose-dependency of *B. anthracis *arginase activity using viable cells

**Figure 5:**
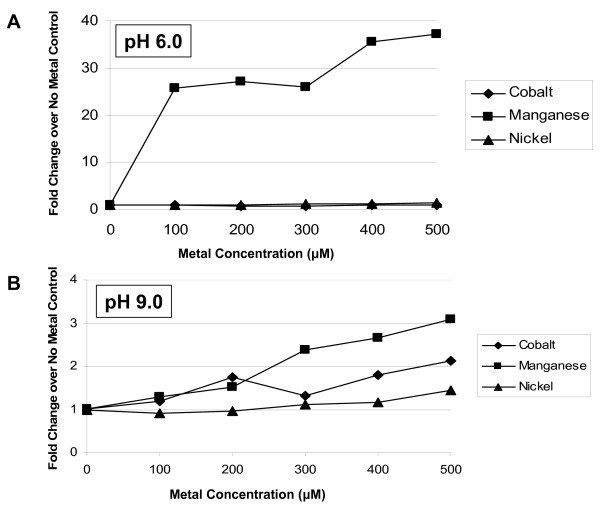
**Effect of metal concentration on arginase activity in *E. coli *(pBS-*barocF*) whole cells**. Cells were grown in the absence or presence of various concentrations of metal (CoCl_2_, MnSO_4_, or NiCl_2_). No effect on growth was observed at the concentrations used. Whole cells were harvested and assayed directly without heat treatment (ice, 30 min) at pH 6.0 (A) or 9.0 (B) using MES- or Tris-arginine buffer, respectively. Data shown is represented as the fold increase over the no metal control. Under these conditions, *E. coli *DH5α(pBS) showed a background of ~1000 pmol L-orn/min/mg protein [U]. Before fold-increases were calculated, 1000 U was subtracted from each *E. coli *DH5α (pBS-*barocF*) data point.

**Figure 6:**
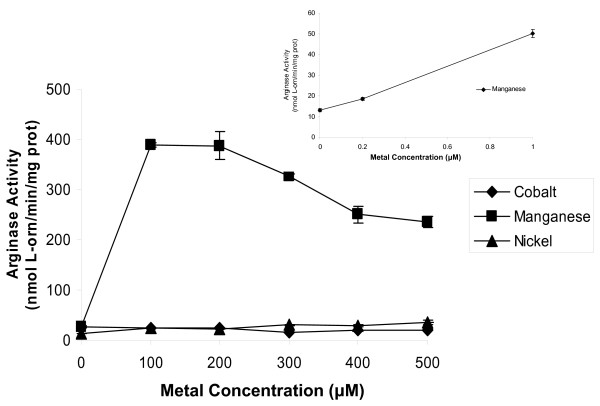
**Evidence that *B. anthracis *7702 has a high affinity and specificity manganese transporter involved in arginase activity**. *B. anthracis *7702 were grown in BHI broth in the absence or presence of metal (CoCl_2_, MnSO_4_, NiCl_2_) at various concentrations. No effect on growth was observed at the concentrations used. Cells were harvested and assayed directly (viable whole cells) without heat treatment (ice, 30 min) at pH 9.0 using BTP-buffered arginine. Inset. *B. anthracis *grown with 0.2 to 1.0 μM MnSO_4_.

The *B. anthracis *arginase characteristics in live cells was examined to better ascertain whether the *in vitro *biochemical results would hold true in viable cells. Previously, we showed that *E. coli *(pBS-*rocF*) increases its arginase activity when grown with increasing concentrations of metal [11]. Here, we determined whether arginase could be detected in viable cells of *B. anthracis *or in *E. coli *(pBS-*barocF*), and if so, what metal was ideal for catalytic activity. In both viable cell models, arginase activity was detectable (Fig. 5 and 6). At pH 6.0 with *E. coli *(pBS-*barocF*), growing the cells in the presence of manganese (≥ 100 μM) increased arginase activity of viable cells (Fig. 5A); cobalt and nickel had no significant effect. At pH 9.0, there was evidence of a slight dose-dependent increase with cobalt, nickel, or manganese (Fig. 5B), with manganese showing the greatest effect.

These experiments were also conducted in *B. anthracis*. Remarkably, arginase activity increased when the bacteria were grown with exceptionally low manganese concentrations (200 nM; Fig. 6 inset). Even at the highest level of cobalt or nickel that can be used without adversely affecting growth (500 μM for cobalt; 1 mM for nickel), cobalt or nickel had no effect on arginase activity (Fig. 6).

### Disruption of the *B. anthracis rocF *in pBS-*barocF *abolishes arginase activity in *E. coli*

Above, it was demonstrated that *B. anthracis rocF *confers arginase activity to *E. coli*, suggesting that *rocF *is necessary and sufficient to confer arginase activity to *E. coli*. Further proof was obtained by disrupting the *rocF *gene in pBS-*barocF *and transforming the resultant plasmid, pBS-*barocF::kan*, into *E. coli *to yield two transformants. One transformant had the kanamycin cassette in the forward (F) orientation with respect to *rocF*, while the other had the cassette in the reverse (R) orientation. *E. coli *carrying either clone had no detectable arginase activity (0.84 ± 0.06 nmol L-ornithine/min/mg prot [U] for pBS-*barocF::kan *F and 0.73 ± 0.2 U for pBS-*barocF::kan *R), in contrast with pBS-*barocF *(3,320 ± 360 U) (heat-activated with manganese, BTP-arginine buffer, pH 9.0). *E. coli *carrying the insert-free vector control, pBS, had 0.57 ± 0.03 U of background activity under these conditions.

### Characteristics of purified *B. anthracis *RocF

**Figure 7:**
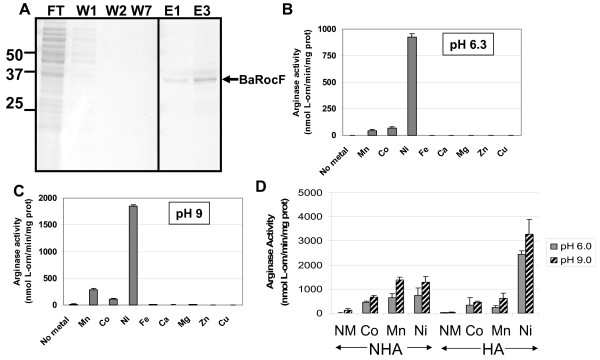
**Characteristics of purified *B. anthracis *His_6_-BaRocF**. **A. Purification of His_6_-BaRocF**. His_6_-BaRocF was purified as described in Methods and the flow-through (FT), washes (W) and elutions (E) analyzed by Coomassie blue-stained SDS-PAGE. Intervening lanes between W7 and E1 have been cropped out. **B and C. Purified His_6_-BaRocF displays optimal catalytic activity with nickel**. The purified enzyme was assayed with 5 mM of the appropriate metal after heat activation at pH 6.3 **(B) **or pH 9.0 **(C) **in Bis Tris Propane [BTP]-buffered arginine. At least two experiments were conducted for each sample in triplicate. Data are presented as mean arginase activity (nmol L-orn/min/mg protein) ± standard deviation. No Metal, assayed without metal. **D. Effect of heat activation on the activity of His_6_-BaRocF**. The purified arginase samples were assayed with or without heat activation (50–55°C or on ice for 30 minutes, respectively) in the absence (No Metal) or presence of 5 mM metal (CoCl_2_, MnSO_4_, or NiCl_2_) at pH 6.0 (in MES-buffered arginine) or 9.0 (Tris-buffered arginine). There was no evidence of a buffer effect on activity (data not shown). At least two experiments were conducted in triplicate for each sample. Data are presented as mean arginase activity (nmol L-orn/min/mg protein) ± standard deviation.

To determine whether the experimental results with *B. anthracis *and the *E. coli *model also hold true for the purified protein, we constructed an *E. coli *strain carrying the *B. anthracis rocF *gene translationally-fused to a his_6 _tag at the N-terminus (designated His_6_-BaRocF). Previous results indicated that the six histidine tag did not interfere with arginase activity [11]. The *B. anthracis *His_6_-BaRocF protein was purified to >95% homogeneity (Fig. 7A). Purified His_6_-BaRocF had a K_m _for arginine of 10 mM and a V_max _of 500 pmol/sec at pH 9.0 in the presence of 5 mM nickel.

When purified His_6_-BaRocF samples were assayed in the presence of nickel, the activity was up to 100-fold higher than with manganese or cobalt (Fig. 7B, 7C), similar to the findings in *B. anthracis *extracts (Fig. 1). Little to no arginase activity occurred in the presence of other divalent cations. Additionally, the metal preference shifted from Ni > Co > Mn at pH 6.3 to Ni > Mn > Co at pH 9.0, as was observed in the *E. coli *model (Fig. 4A).

In a previous study, *B. anthracis *arginase activity decreased in samples exposed to heat (50–60°C), if the enzyme had not been pre-activated with manganese [23]. We determined that in the presence of cobalt, heat activation did not adversely affect arginase activity at pH 6.0 or 9.0, when compared to samples that were not heat-treated (Fig. 7D). Remarkably, heat treatment dramatically increased arginase activity in the presence of nickel at either pH 6.0 or 9.0. As observed in *B. anthracis *extracts and in extracts using the *E. coli *model, optimal catalytic activity was observed when the purified enzyme was assayed in the presence of nickel (Fig. 7D), rather than manganese or cobalt.

**Figure 8:**
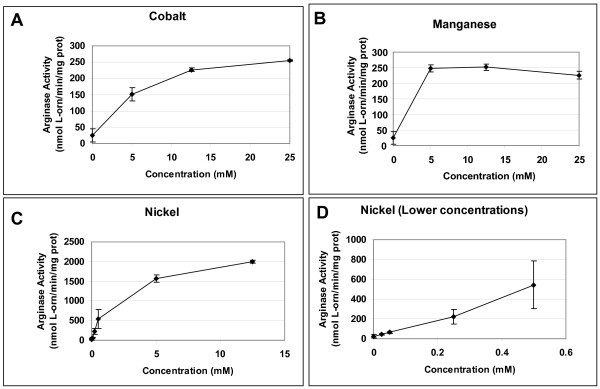
**His_6_-BaRocF assayed in the presence of various concentrations of different metals**. The purified arginase samples were heat activated (50–55°C for 30 minutes) with increasing concentrations of **(A) **CoCl_2_, **(B) **MnSO_4_, or **(C, D) **NiCl_2 _at pH 9.0 (in BTP-buffered arginine). At least two experiments were conducted for each sample in triplicate. Data are presented as mean arginase activity (nmol L-orn/min/mg protein) ± standard deviation.

Catalytic activity of purified *B. anthracis *arginase was determined at various concentrations of nickel, manganese, or cobalt (Fig. 8). Nickel showed an increase in activity at a much lower concentration when compared to manganese or cobalt, confirming the results found in extract samples from the native organism and in *E. coli *expressing the *B. anthracis *arginase. Micromolar nickel concentrations activated arginase >2-fold more than the optimal level of manganese (544 nmol L-orn/min/mg prot [U] versus 251 U), similar to evidence from extract samples. Additionally, optimal concentrations for the three metals were: nickel, ≥ 12.5 mM; manganese, ≥ 5 mM; and cobalt, 25 mM (Fig. 8). At these optimal concentrations of metal, nickel conferred eight times more arginase activity than either manganese or cobalt.

### Complementation of the *H. pylori *arginase mutant with the *B. anthracis rocF *gene

**Figure 9:**
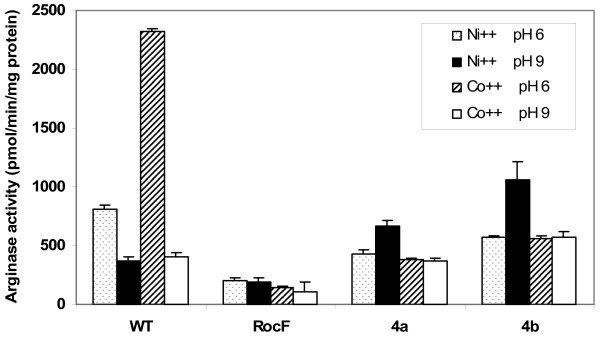
**Complementation of the *H. pylori rocF *mutant with the *B. anthracis rocF *gene**. Cellular extracts from wild type *H. pylori *26695 (WT), the 26695 *rocF *mutant (RocF) and the *rocF *complemented with the *B. anthracis rocF *gene (clones 4a and 4b) were assayed for arginase activity at pH 6.0 (MES-arginine buffer) or 9.0 (Tris-arginine buffer) in the presence of cobalt or nickel with heat activation (50°C for 30 minutes). Representative of two experiments conducted in triplicate.

Since we were unsuccessful in several attempts to disrupt the *rocF *gene in *B. anthracis*, we instead transformed the *B. anthracis rocF *gene into an *H. pylori rocF *mutant devoid of arginase activity. This was achieved using the suicide plasmid complementation system developed previously [18], but with an improved vector backbone, pLSU0005 (see Methods). Expression of the *B. anthracis rocF *gene was driven off the strong *H. pylori *urease promoter. Transformation of the 26695 *rocF *mutant with the empty vector control was previously shown not to complement arginase activity, as expected [18]. *H. pylori *complemented clones 4a and 4b acquired arginase activity that exhibited *B. anthracis *properties, namely, optimal arginase activity at pH 9 with nickel (Fig. 9), but the overall activity was substantially lower than what is observed for native *B. anthracis*.

## Discussion

In this study arginase from *B. anthracis *was investigated under a variety of variables using six different models: i) extracts from *B. anthracis*; ii) extracts from *E. coli *(pBS-*barocF*); iii) viable cells of *B. anthracis*; iv) viable cells of *E. coli *(pBS-*barocF*); v) purified *B. anthracis *RocF (as His_6_-BaRocF); and vi) extracts from the *H. pylori rocF *complemented with *B. anthracis rocF*. In nearly every case, all models lead to the same conclusions. These conclusions are: i) *B. anthracis *arginase displays highest catalytic activity with nickel as the metal cofactor, when extracts or the purified protein is examined; ii) *B. anthracis *arginase is active without heat activation and without addition of metals, although cobalt, manganese, and nickel all improve activity; iii) the enzyme has much higher activity at pH 9.0 than at pH 6.0; iv) a viable cell arginase assay was developed in both *B. anthracis *and in an *E. coli *model and the characteristics of the enzyme mostly overlapped in the two models; v) viable cells exhibit far greater arginase activity when grown with manganese than with nickel or cobalt; vi) the *B. anthracis *arginase displays up to 1000-fold more specific activity than the *H. pylori *enzyme, depending on the conditions; vii) the *B. anthracis rocF *gene complements an *H. pylori rocF *mutant and confers *B. anthracis*-like arginase properties; and viii) the *B. anthracis *arginase gene is necessary and sufficient to confer arginase activity to *E. coli*.

Most bacteria that possess arginase have a complete urea cycle for eliminating excess nitrogen as urea. In striking contrast, *B. anthracis *contains arginase, but lacks the other enzymes of the urea cycle [23]. The role of arginase in *B. anthracis *pathogenesis and cellular physiology is only recently becoming understood [15,27], but our knowledge is far from complete. This current study uncovered novel characteristics about the enzyme as expressed in *E. coli *and in *B. anthracis*, and therefore lays important groundwork for future experiments in these areas. For the first time, it is demonstrated that the *rocF *gene from *B. anthracis *is the gene responsible for arginase activity, since *E. coli *containing *B. anthracis rocF *possessed arginase activity, while a kanamycin insertion into *rocF *abolished arginase activity. Thus, *rocF *was necessary and sufficient to confer arginase activity to *E. coli*. These results do not, however, rule out the possibility that *B. anthracis *contains genes that influence arginase expression or activity. Indeed, the *B. anthracis *genome contains a homologue of RocR, a positive transcriptional activator of the arginase-containing operon in *B. subtilis *[21].

Based on previous studies using the purified *B. anthracis *arginase [23], it was expected that the enzyme would be most active with manganese as its metal cofactor. In contrast, we identified nickel as the optimum metal cofactor for arginase activity in *B. anthracis *extracts, with purified His_6_-BaRocF, and with extracts from *E. coli *(pBS-*barocF*). Peak *B. anthracis *arginase activity was reached with much lower nickel concentrations than with manganese or cobalt. This suggests that in a cell-free system, the arginase has highest activity with nickel. The reason for the differences between our studies and those published previously [23] may be the use of an improved enzyme assay, coupled with our ability to assay the enzyme in multiple model systems that were unavailable 25 years ago. To our knowledge, this is the first arginase to have optimal activity with nickel. However, it is recognized that the actual metal found *in vivo *in *B. anthracis *native arginase is critical to study in the near future by inductively coupled plasma-mass spectrometry analysis. Such work cannot be accomplished with the current purified protein due to presence of the His_6 _tag and use of a nickel-containing column to purify the protein.

*B. anthracis *arginase was active with cobalt, manganese, and nickel. Therefore, depending on the pH and growth conditions, *B. anthracis *arginase can promiscuously utilize various metals to achieve catalytic activity. This metal promiscuity may allow the arginase to utilize the metal that is most readily available under a given environmental situation or *in vivo *condition encountered by *B. anthracis*. The only other reported bacterial arginase that has good activity with multiple metals is *B. caldovelox *(Table 1) [28], which has an arginase that is active with manganese, cobalt, cadmium and nickel. However, the *B. caldovelox *arginase protein (NCBI Accession Number: S68863), which is 73% identical to *B. anthracis *RocF, has optimal activity with manganese [28].

**Table 1:** Summary of arginase properties from *Bacillus spp*. and *H. pylori*.

**Organism and putative metal-binding site^a^**	**Urea cycle**	**pH optimum**	**Metal optimum**	**Regulation**	**Activity without metal**	**Reference(s)**
*H. pylori *LYLDAHADIHT	Yes	6	Co^2+^	Unknown	No	[11, 20], This study
*B. anthracis *IWYDAHGDLNT	No	9	**Extracts:** Ni^2+ ^> Mn^2+ ^> Co^2+ ^(pH 9.0), Ni^2+ ^> Co^2+ ^> Mn^2+ ^(pH 6.0) **Whole Cells:** Mn^2+ ^>> Ni^2+ ^= Co^2+^	Unknown	Yes	[23], This study
*B. caldovelox *IWYDAHGDVNT	Unknown	9–11	Mn^2+^, Ni^2+^, Co^2+^, Cd^2+^	Unknown	Yes	[17, 28]
*B. licheniformis *IWYDAHGDLNT	Unknown	9.5–10.0	Mn^2+^	Oxygen, arginine, ornithine, proline, urea, glutamine, sporulation: all (+); glucose (-)	Yes	[19, 22, 26, 34, 39]
*B. subtilis *IWYDAHGDLNT	Yes	9–11	Mn^2+^	RocR (+), AhrC (+); arginine, ornithine, citrulline, proline (all+)	Yes	[21, 40, 41]

^a ^Underlined region denotes amino acids most likely involved in metal binding.

In viable cells we showed that arginase activity can be directly assayed from *E. coli *expressing *B. anthracis rocF *or in native *B. anthracis*. This implies that the substrate, L-arginine, was transported into the cells, and was converted to urea and L-ornithine; the latter was measured in this study. Viable *B. anthracis *cells have up to ten-fold higher arginase activity with manganese than with cobalt or nickel (Fig. 6). This finding seemingly contradicts our earlier finding that the *B. anthracis *arginase in cell-free systems has highest activity with nickel. Two possible interpretations of this discrepancy are that i) *B. anthracis *arginase actually uses manganese rather than nickel *in vivo *and ii) *B. anthracis *arginase uses nickel, manganese or cobalt, depending on the situation. Since viable *B. anthracis *displayed a remarkable increase in arginase activity when the organisms were grown with extremely low levels of manganese (200 nM), we propose that *B. anthracis *expresses a very high affinity and specificity manganese transporter under our experimental conditions; this transporter would not be expected to transport nickel or cobalt, since addition of these metals did not increase *B. anthracis *arginase activity in viable cells (Fig. 6). It is speculated that such a transporter would be able to scavenge manganese from sites expected to be very low in manganese concentrations, such as would exist *in vivo*. Additional experiments are warranted to directly determine the metal cofactor found in the *B. anthracis *arginase active site.

The heat-activation step, which involves heating arginase at 50–60°C in the presence of metal, is required to obtain arginase activity in *H. pylori *and many other previously characterized arginases [11,24,26,29-31]. Previous studies have shown that heat-activation decreases *B. anthracis *arginase activity if manganese is absent [23]; we observed this with the purified protein as well. In contrast, we found that heating purified arginase in the presence of nickel dramatically improves activity over that of arginase that had not been heat-activated (Fig. 3). This result suggests that arginase may fold in a more ideal conformation with heat treatment in the presence of nickel than with cobalt or manganese, raising the possibility that nickel is the physiologic metal cofactor used by *B. anthracis *arginase. Despite these observations, however, substantial *B. anthracis *arginase activity still remains without heat-activation (Fig. 3, 8), unlike the case for most previously characterized arginases. Moreover, *B. anthracis *arginase has substantial activity without exogenously added metal (Fig. 3, 4), suggesting that sufficient levels of the residual metal cofactor may already be tightly bound to the enzyme's active site. This raises the possibility that the metal cofactor is more tightly bound to *B. anthracis *RocF than it is in other arginases. All of the other arginase have been proposed to have at least one of the two metal ions loosely bound to the active site [24,32,33].

We demonstrated that the *H. pylori rocF *mutant devoid of arginase activity could regain arginase activity with *B. anthracis*-like properties when the *rocF *mutant was complemented with the *B. anthracis rocF *gene (Fig. 9), suggesting that the *B. anthracis *RocF protein itself harbors at least some of the properties that allow it to have optimal activity with nickel. However, the *B. anthracis rocF *gene did not fully restore arginase activity to *H. pylori *even though a strongly active *H. pylori *urease promoter was used. This lack of full restoration may be due to codon usage differences between *H. pylori *and *B. anthracis *or to species-specific proteins that affect arginase activity. For example, *H. pylori *may have an arginase metal-delivering chaperone that does not recognize the *B. anthracis *enzyme. It is also possible that *H. pylori *has mechanisms to prevent arginase activity from becoming too high, which could lead to arginine starvation, since it was previously shown that wild type *H. pylori *carrying two copies of the arginase gene only have 25% more arginase activity than the wild type strain with a single copy [18]. Arginine is an essential amino acid for *H. pylori *but not *B. anthracis*. *B. anthracis *can therefore have much higher arginase activity without starving itself for arginine, since *B. anthracis *can synthesize more arginine intracellularly.

Table 1 summarizes the properties of a subset of eubacterial arginases. It is readily apparent that even within the genus *Bacillus*, whose arginases range from 66–99% identical at the amino acid level to *B. anthracis*, unique arginase properties exist. For example, multiple metals can be used for the arginase of *B. caldovelox *[28], while sporulation induces arginase in *B. licheniformis *[34], and the exosporium of spores from *B. anthracis *contain arginase activity [27]. While the putative metal-binding region is nearly completely conserved among the *Bacillus *species, there are a number of differences compared to the *H. pylori *arginase that may serve as important residues to target for site-directed mutagenesis.

## Conclusion

The *H. pylori *arginase displays unique pH (6.0) and metal (cobalt) optima within the arginase superfamily. From the results of this study, the *B. anthracis *arginase likely displays a unique metal (nickel) optimum in the arginase superfamily and can potentially use multiple metals (cobalt, nickel, or manganese) to achieve catalytic activity suitable for its niche. Characteristic differences in arginases of *B. anthracis *versus *H. pylori *may reflect distinct *in vivo *niches occupied by these organisms. This study provides the foundation for further examination of the role of arginase in *B. anthracis *pathogenesis and cellular physiology.

## Methods

### Bacterial strains, growth conditions, and plasmids

*Escherichia coli *strain DH5α [F^-^, *deoR*, *thi-1*, *gyrA96*, *recA1*, *endA1*, *relA1*, *supE44*, Δ (*lacZYA*-*argF*) *U169*, *hsdR17 *(*r*^-^, *m*^+^), *φ *80d *lacZΔM15*, λ^-^] (Stratagene, La Jolla, CA) was used for standard cloning and transformation procedures. Strain XL1-Blue MRF' [F-, *thi-1, gyrA96, recA1, endA1, relA1, supE44, lac, hsdR17 (r^-^, m^+^), F' [proAB, lacI^q^Z)M15, Tn10 [tet^r^]*] *E. coli *was grown on Luria (L) agar and in L broth plus appropriate antibiotics (ampicillin, 100 μg/mL; kanamycin, 25 μg/mL; tetracycline 15 μg/mL) at 37°C for ~24 hours; if grown in broth, the cultures were aerated (225 rpm).

*Bacillus anthracis *Sterne toxigenic acapsulate strain 7702 (pXO1^+^, pXO2^-^) [7,35] was grown in Brain Heart Infusion (BHI) broth, L broth, or on Campylobacter agar with 10% (v/v) defibrinated sheep blood (CBA) at 37°C for ~24 hours in 5% CO_2 _in air or in 10% CO_2_, 5% O_2_, 85% N_2_; when grown in broth, cultures were aerated (225 rpm).

*Helicobacter pylori *43504 was grown from frozen stocks for 72 hours on CBA at 37°C, passaged to fresh CBA for an additional 48 hours and then transferred to 25 mL Ham's F-12 plus 2% fetal bovine serum (HyClone, Logan, UT) and incubated for 24 hours at 37°C. All conditions were microaerobic (10% CO_2_, 5% O_2_, 85% N_2_). The bacteria were then centrifuged for 10 min (6,000 × *g*) and resuspended in 600 μL 0.9% NaCl. Aliquots (100 μL) were spread onto 6 CBA plates and incubated at 37°C for 24 hours. The resultant cultures were used in the assays as described below.

Plasmid pBS [pBluescript II SK (+), Stratagene], pBS-*rocF *[11], and pBS-*barocF *(see below) were used in this study.

### Molecular biology techniques

Plasmid DNA was isolated by the alkaline lysis method [36], or by using a column chromatography kit (Qiagen, Valencia, CA) for sequencing-grade plasmid. Restriction endonuclease digestions, ligations, and other enzyme reactions were conducted according to the manufacturer's instructions (Promega, Madison, WI or New England Biolabs, Beverly, MA). PCR reactions (50 μL) contained 10 to 100 ng of DNA, PCR buffer, 2.0 to 2.5 mM MgCl_2_, dNTPs (each nucleotide at a concentration of 0.20 to 0.25 mM), 200 pmol of each primer, and 2.5 units of thermostable DNA polymerase. *E. coli *was transformed by the calcium chloride method or electroporation.

### Cloning of *B. anthracis rocF *into pBS

The *B. anthracis *arginase gene, *rocF *(1104 bp), including the 893 bp coding region, 184 bp upstream, and 27 bp downstream was PCR-amplified from the chromosome using primers DM55(5'-cgggatccTTAATAATAATGATGGTAGTTGCTTCA-3') and DM56(5'-ccatcgatGCAACTTCTCAGTTGCTTTTCTTACAT-3') [uppercase letters: *rocF *gene; lowercase letters: *Bam*HI (underlined) on the forward primer or *Cla*I (underlined) on the reverse primer]. The PCR product was digested with *Bam*HI and *Cla*I and cloned into pBS digested with the same enzymes to generate pBS-*barocF*. The construct was confirmed by sequence analysis, restriction enzyme digestion (data not shown), and enzyme activity (see below). *B. anthracis *RocF, with a predicted mass of 32.7 kDa, was shown to be expressed by sodium dodecyl sulfate-polyacrylamide gel electrophoresis (SDS-PAGE) analysis (data not shown).

### Construction of pBS-*barocF::kan*

To disrupt the *B. anthracis *arginase gene, the plasmid pBS-*kan *[37] was digested with *Sma*I and *Hinc*II to generate the blunt-ended non-polar kanamycin resistance cassette (~1.3 kb), and pBS-*barocF *was digested with *Hind*III (~4.2 kb). The pBS-*barocF *linearized fragment was rendered blunt-ended by Klenow and ligated to the kanamycin cassette using the Roche Rapid DNA Ligation Kit (Roche Diagnostics Corporation, Indianapolis, IN). The ligation mixture was transformed into *E. coli *DH5α and doubly antibiotic resistant (kanamycin, ampicillin) transformants were verified by restriction digestion and arginase assay.

### Construction of *E. coli *XL1-Blue MRF' (pQE30-*barocF*), overexpressing *B. anthracis *RocF

Plasmid pQE30 was digested with *Bam*HI and *Kpn*I, purified using the QIAquick Gel Extraction Kit (Qiagen, Valencia, CA). The DNA was dephosphorylated using shrimp alkaline phosphatase. The *B. anthracis rocF *coding region was amplified from the chromosome using DM184, 5'-GGggatccAAAAAAGAAATTTCGGTT-3' (with non-*B. anthracis *sequence designated as underlined capital letters and the *Bam*HI site in lowercase letters) and DM67, 5'-GGggtaccCCTTTTAGTTTTTCACCGAATA-3'(with non-*B. anthracis *sequence designated as underlined capital letters and the *Kpn*I site in lowercase letters). The ~900 bp PCR product was cloned into *E. coli *TOP10 using the pCR2.1 vector according to the manufacturer's instructions (TOPO TA Cloning Kit, Invitrogen Life Technologies, Carlsbad, CA 92008). After digestion with *Bam*HI and *Kpn*I, the *rocF *gene was purified from the vector by agarose gel electrophoresis and ligated to the digested, phosphatase-treated pQE30 and transformed into XL1-Blue MRF' to yield pQE30-*barocF*. Transformants (amp^r^, tet^r^) were confirmed via restriction digestion analysis, PCR analysis, and arginase activity (data not shown).

### Complementation of the *H. pylori *arginase mutant with the *B. anthracis rocF *gene

The *B. anthracis rocF *coding region was PCR-amplified from pBS-*barocF *using DM278 (5'-GCGCTGCAGGGATGAAAAAAGAAATTTCGG-3' and DM279 (5'-GCCCATGGCTTCTCAGTTGCTTTTCTTAC-3'). The ~900 bp product was digested with *Pst*I and *Nco*I, and cloned downstream of the strong *H. pylori *urease promoter in pLSU0005 to yield pW7. Plasmid pLSU0005 is a derivative of suicide plasmid pIR203C04 [18] that has an improved multi-cloning site, the *ureA *promoter and a chloramphenicol resistance cassette and is used for complementation in *H. pylori*. Plasmid W7 (5 μg) was electroporated into the *rocF *mutant of *H. pylori *26695 [38] and two chloramphenicol resistant transformants were confirmed by PCR analysis using flanking primers (data not shown); clones 4a and 4b were subsequently used.

### Purification of the *Bacillus anthracis *arginase

*E. coli *XL1-Blue MRF' (pQE30-*barocF*) was grown overnight, diluted 1:100 in 500 mL of L broth plus ampicillin and tetracycline and the culture was incubated at 37°C, 225 rpm for 3–5 h (OD_600 nm _= ~0.7). Isopropyl thio-β-D-galactopyranoside (2.5 mM, final concentration) was added and the culture incubated for an additional 3–5 h at 37°C, 225 rpm. The resulting culture was centrifuged at 5,000 rpm (Sorval, SH-3000) for 15 min, 4°C. The supernatant was discarded and the pellet was resuspended in 1/20^th ^to 1/40^th ^the original culture volume in Wash/Lysis buffer (50 mM NaH_2_PO_4_, 300 mM NaCl, 15 mM imidazole, pH 8.0). The bacteria were lysed by two passages through a French Press (16,000 psi) and maintained at 4°C throughout the remainder of the purification procedure. The lysates were then centrifuged and the supernatant containing arginase activity was retained. For every 680 μL of cytosol, 320 μL of nickel-nitrilotriacetic acid/ethanol agarose resin (Ni-NTA, Qiagen) was added. This solution was gently mixed end-over-end for 1–2 h at 4°C. The cytosol/Ni-NTA agarose mixture was equally distributed among two, 8.5 by 2.0 cm polypropylene columns. The flow through was reserved in sterile polypropylene test tubes, and the columns were washed 6–8 times with ~10 mL Wash/Lysis buffer per wash. These washes were retained for SDS-PAGE analysis. The protein was eluted using Elution Buffer (50 mM NaH_2_PO_4_, 300 mM NaCl, 250 mM imidazole, pH 8.0) at 1 mL per elution, for a total of 12 elutions per column. The flow through fractions, washes, and elutions were analyzed for protein by SDS-PAGE, and the elutions were monitored for arginase activity (data not shown). All samples were stored at 4°C or at -20°C in 50% glycerol.

### Preparation of arginase-containing extracts

Bacteria were harvested from solid medium using a sterile swab and resuspended in 0.9% NaCl or phosphate-buffered saline (PBS). Broth-grown cells were centrifuged at 16,000 × *g *for 5 minutes. The pelleted cells were resuspended in 150 μl of 0.9% NaCl or PBS. For whole cell assays, bacteria were directly assayed. For extracts, the suspensions were sonicated (25% intensity, Sonic Dismembrator Model 500, Fisher Scientific, Pittsburgh, PA) in an ice bath twice for 30 sec with a minimum 30 sec rest on ice between pulses. Following sonication, the lysate was clarified by centrifugation at 16,000 × *g *for 5 minutes. The resulting supernatant was retained on ice and used the same day for determination of arginase activity.

### Arginase Assay

Extracts or viable bacteria were characterized using the standard arginase assay described previously [11] or with variations (described below). The sample (25 μl) was added to 25 μL of CoCl_2_, MnSO_4_, NiCl_2_, FeSO_4_, CaCl_2_, MgSO_4_, ZnCl_2_, or CuSO_4 _(final concentration of 5 mM, except as noted) or 25 μL of deionized distilled H_2_O (ddH_2_O, no metal control). An L-ornithine (0 to 3125 μM) standard curve was generated (extinction coefficient was typically 0.00045 to 0.00070 μM^-1^). The samples and standards were heat-activated (50–55°C, 30 min) or were maintained on ice for 30 min. Next, 200 μL of buffered 10 mM L-arginine (15 mM MES [2-(N-morpholino)ethanesulfonic acid], pH 6.0; 15 mM Tris, pH 9.0; or 15 mM Bis-Tris Propane [BTP], pH 6.3 or 9.0) was added, and the samples were incubated at 37°C for 1 hour. Enzyme rates were linear at this time point. The reaction was stopped by the addition of acidified ninhydrin (4 mg/mL). After heating for one hour at 90–95°C, the standards and samples were measured spectrophotometrically at 515 nm (Biomate 3, Thermo Spectronic, Rochester, NY) in 1.5 ml cuvettes. Representative data were normalized for protein and are presented as specific activity in pmol or nmol L-ornithine/min/mg protein ± standard deviation with a minimum of two experiments conducted in duplicate or triplicate. Some graphs were converted to log scale since the enzyme activity is much higher under certain experimental conditions. Some experiments were performed using BTP as a buffer, because it had a broad buffering capacity of pH 6.3 to 9.5, which would eliminate any potential buffer effects. It was determined that biochemical intermediates and end products, such as putrescine, spermidine, spermine and urea, did not react with ninhydrin (data not shown).

### Protein determinations

Protein determinations were performed by the Bicinchoninic Acid assay (Pierce Chemical Company, Rockford, IL), following the manufacturer's 30 minute method. NaCl or PBS was used as the negative control. The results were calculated by a standard curve using bovine serum albumin.

### SDS-PAGE analysis

Proteins were electrophoresed through an SDS-polyacrylamide gel (12%) by standard methods.

### Statistical Analyses

An unpaired two-tailed Welch's *t *test was used to determine statistical relationships (GraphPad Instat 3.05). *p *< 0.05 was considered significant.

## Authors' contributions

RJV co-designed and conducted nearly all of the experiments and drafted the manuscript and figures in partial fulfillment of requirements for the Masters degree. EH conducted the experiments on complementation of *H. pylori *arginase with the *B. anthracis rocF*; RFR provided scientific advice and assisted with the writing; DJM directed the project and edited the manuscript.

## References

[B1] Koehler TM, Dai Z, Kaufman-Yarbray M (1994). Regulation of the Bacillus anthracis protective antigen gene: CO2 and a trans-acting element activate transcription from one of two promoters. J Bacteriol.

[B2] Fish DC, Mahlandt BG, Dobbs JP, Lincoln RE (1968). Purification and properties of in vitro-produced anthrax toxin components. J Bacteriol.

[B3] Leppla SH (1984). Bacillus anthracis calmodulin-dependent adenylate cyclase: chemical and enzymatic properties and interactions with eucaryotic cells. Adv Cyclic Nucleotide Protein Phosphorylation Res.

[B4] Stanley JL, Smith H (1961). Purification of factor I and recognition of a third factor of the anthrax toxin. J Gen Microbiol.

[B5] Beall FA, Taylor MJ, Thorne CB (1962). Rapid lethal effect in rats of a third component found upon fractionating the toxin of Bacillus anthracis. J Bacteriol.

[B6] Smith H, Stoner HB (1967). Anthrax toxic complex. Fed Proc.

[B7] Shannon JG, Ross CL, Koehler TM, Rest RF (2003). Characterization of anthrolysin O, the Bacillus anthracis cholesterol-dependent cytolysin. Infect Immun.

[B8] Davis RH (1986). Compartmental and regulatory mechanisms in the arginine pathways of Neurospora crassa and Saccharomyces cerevisiae. Microbiol Rev.

[B9] Green SM, Eisenstein E, McPhie P, Hensley P (1990). The purification and characterization of arginase from Saccharomyces cerevisiae. J Biol Chem.

[B10] Sumrada RA, Cooper TG (1984). Nucleotide sequence of the Saccharomyces cerevisiae arginase gene (CAR1) and its transcription under various physiological conditions. J Bacteriol.

[B11] McGee DJ, Zabaleta J, Viator RJ, Testerman TL, Ochoa AC, Mendz GL (2004). Purification and characterization of Helicobacter pylori arginase, RocF: unique features among the arginase superfamily. Eur J Biochem.

[B12] Read TD, Peterson SN, Tourasse N, Baillie LW, Paulsen IT, Nelson KE, Tettelin H, Fouts DE, Eisen JA, Gill SR, Holtzapple EK, Okstad OA, Helgason E, Rilstone J, Wu M, Kolonay JF, Beanan MJ, Dodson RJ, Brinkac LM, Gwinn M, DeBoy RT, Madpu R, Daugherty SC, Durkin AS, Haft DH, Nelson WC, Peterson JD, Pop M, Khouri HM, Radune D, Benton JL, Mahamoud Y, Jiang L, Hance IR, Weidman JF, Berry KJ, Plaut RD, Wolf AM, Watkins KL, Nierman WC, Hazen A, Cline R, Redmond C, Thwaite JE, White O, Salzberg SL, Thomason B, Friedlander AM, Koehler TM, Hanna PC, Kolsto AB, Fraser CM (2003). The genome sequence of Bacillus anthracis Ames and comparison to closely related bacteria. Nature.

[B13] Nathan C, Shiloh MU (2000). Reactive oxygen and nitrogen intermediates in the relationship between mammalian hosts and microbial pathogens. Proc Natl Acad Sci U S A.

[B14] Gobert AP, McGee DJ, Akhtar M, Mendz GL, Newton JC, Cheng Y, Mobley HL, Wilson KT (2001). Helicobacter pylori arginase inhibits nitric oxide production by eukaryotic cells: a strategy for bacterial survival. Proc Natl Acad Sci U S A.

[B15] Raines KW, Kang TJ, Hibbs S, Cao GL, Weaver J, Tsai P, Baillie L, Cross AS, Rosen GM (2006). Importance of nitric oxide synthase in the control of infection by Bacillus anthracis. Infection & Immunity.

[B16] Zabaleta J, McGee DJ, Zea AH, Hernandez CP, Rodriguez PC, Sierra RA, Correa P, Ochoa AC (2004). Helicobacter pylori arginase inhibits T cell proliferation and reduces the expression of the TCR z-chain (CD3z). J Immunol.

[B17] Bewley MC, Jeffrey PD, Patchett ML, Kanyo ZF, Baker EN (1999). Crystal structures of Bacillus caldovelox arginase in complex with substrate and inhibitors reveal new insights into activation, inhibition and catalysis in the arginase superfamily. Structure Fold Des.

[B18] Langford ML, Zabaleta J, Ochoa AC, Testerman TL, McGee DJ (2006). In Vitro and In Vivo Complementation of the Helicobacter pylori Arginase Mutant Using an Intergenic Chromosomal Site. Helicobacter.

[B19] Cunin R, Glansdorff N, Pierard A, Stalon V (1986). Biosynthesis and metabolism of arginine in bacteria. Microbiol Rev.

[B20] Mendz GL, Hazell SL (1996). The urea cycle of Helicobacter pylori. Microbiology.

[B21] Gardan R, Rapoport G, Debarbouille M (1997). Role of the transcriptional activator RocR in the arginine-degradation pathway of Bacillus subtilis. Mol Microbiol.

[B22] Broman K, Lauwers N, Stalon V, Wiame JM (1978). Oxygen and nitrate in utilization by Bacillus licheniformis of the arginase and arginine deiminase routes of arginine catabolism and other factors affecting their syntheses. J Bacteriol.

[B23] Soru E (1983). Chemical and immunological properties of B. anthracis arginase and its metabolic involvement. Mol Cell Biochem.

[B24] Mohamed MS, Greenberg DM (1945). Liver Arginase I. Preparation of extracts of high potency, chemical properties, activation-inhibition, and pH activity.. Arch Biochem Biophys.

[B25] Jenkinson CP, Grody WW, Cederbaum SD (1996). Comparative properties of arginases. Comp Biochem Physiol B Biochem Mol Biol.

[B26] Hirsch-Kolb H, Heine JP, Kolb HJ, Greenberg DM (1970). Comparative physical-chemical studies of mammalian arginases. Comp Biochem Physiol.

[B27] Weaver J, Kang TJ, Raines KW, Cao GL, Hibbs S, Tsai P, Baillie L, Rosen GM, Cross AS (2007). Protective role of Bacillus anthracis exosporium in macrophage-mediated killing by nitric oxide. Infection & Immunity.

[B28] Patchett ML, Daniel RM, Morgan HW (1991). Characterisation of arginase from the extreme thermophile 'Bacillus caldovelox'. Biochim Biophys Acta.

[B29] Greenberg DM, Bagot AE, Roholt OA (1956). Liver arginase. III. Properties of highly purified arginase. Arch Biochem Biophys.

[B30] Ramaley RF, Bernlohr RW (1966). Postlogarithmic phase metabolism of sporulating microorganisms. II. The occurrence and partial purification of an arginase. J Biol Chem.

[B31] Schreier HJ, Smith TM, Bernlohr RW (1982). Regulation of nitrogen catabolic enzymes in Bacillus spp. J Bacteriol.

[B32] Brown GW (1966). Studies in comparative biochemistry and evolution. I. Avian liver arginase. Arch Biochem Biophys.

[B33] Christianson DW (2005). Arginase: structure, mechanism, and physiological role in male and female sexual arousal. Acc Chem Res.

[B34] Simon JP, Stalon V (1976). Purification and structure of arginase of Bacillus licheniformis. Biochimie.

[B35] Cataldi A, Labruyere E, Mock M (1990). Construction and characterization of a protective antigen-deficient Bacillus anthracis strain. Mol Microbiol.

[B36] Sambrook J, Fritsch EF, Maniatis T, Laboratory CSH (1989). Molecular Cloning:  A Laboratory Manual.

[B37] McGee DJ, Coker C, Testerman TL, Harro JM, Gibson SV, Mobley HL (2002). The Helicobacter pylori flbA flagellar biosynthesis and regulatory gene is required for motility and virulence and modulates urease of H. pylori and Proteus mirabilis. J Med Microbiol.

[B38] McGee DJ, Radcliff FJ, Mendz GL, Ferrero RL, Mobley HL (1999). Helicobacter pylori rocF is required for arginase activity and acid protection in vitro but is not essential for colonization of mice or for urease activity. J Bacteriol.

[B39] Laishley EJ, Bernlohr RW (1968). Regulation of arginine and proline catabolism in Bacillus licheniformis. J Bacteriol.

[B40] Baumberg S, Harwood CR (1979). Carbon and nitrogen repression of arginine catabolic enzymes in Bacillus subtilis. J Bacteriol.

[B41] De Hauwer G (1964). Pyrroline dehydrogenase and catabolism regulation of arginine and proline in Bacillus subtilis.. Biochim Biophys Acta.

